# Exploring the clinical characteristics and etiological factors of comorbid major depressive disorder and social anxiety disorder

**DOI:** 10.17305/bb.2023.9690

**Published:** 2023-12-01

**Authors:** Tonguc Demir Berkol, Ipek Özönder Ünal

**Affiliations:** 1 Department of Psychiatry, Bakirkoy Research and Training Hospital for Psychiatry, Neurology and Neurosurgery, Istanbul, Turkey; 2 Department of Psychiatry, Istanbul Tuzla State Hospital, Istanbul, Turkey

**Keywords:** Adjustment disorders, adverse childhood experiences, anxiety, blood cell count, inflammation, major depressive disorder (MDD), social anxiety disorder (SAD), lymphocyte count, monocytes, neutrophils, social phobia

## Abstract

The comorbidity between the major depressive disorder (MDD) and the social anxiety disorder (SAD) is significantly prevalent, necessitating a nuanced understanding of their overlapping clinical characteristics and shared etiological factors, including inflammatory biomarkers. To address this, we conducted a cross-sectional study from December 2021 to June 2022, encompassing 204 outpatients diagnosed with MDD–SAD comorbidity. We employed various psychometric assessments, such as the Beck Depression Inventory (BDI), Beck Anxiety Inventory (BAI), Childhood Trauma Questionnaire (CTQ-28), Toronto Alexithymia Scale (TAS-20), and the Liebowitz Social Anxiety Scale (LSAS). Additionally, we analyzed inflammatory biomarkers, including the neutrophil-to-lymphocyte ratio (NLR), monocyte-to-lymphocyte ratio (MLR), platelet-to-lymphocyte ratio (PLR), neutrophil-to-lymphocyte platelet ratio (NLPR), systemic inflammation index (SII), and the systemic inflammation response index (SIRI). Our findings accentuated that patients primarily diagnosed with MDD exhibited elevated levels of certain inflammatory biomarkers. They reported more severe and atypical depressive symptoms (75.7% vs 58.5%; *P* ═ 0.010) and had significantly higher CTQ-28 subscale scores (*P* < 0.05). Our study unveils a complex relationship between MDD and SAD, with significant disparities in the symptom severity and inflammatory biomarker levels, thereby establishing a compelling case for dual-diagnosis treatment approaches. It elucidates the critical role of inflammation in the comorbidity of MDD and SAD, marking a pioneering step towards more comprehensive and holistic patient care strategies. These insights could potentially revolutionize therapeutic approaches in psychiatric care, promising significantly improved outcomes through early detection and integrated intervention strategies.

## Introduction

In recent decades, society has seen a burgeoning awareness and understanding of mental health disorders, a term that encapsulates a wide range of conditions affecting millions globally. Within this sphere, anxiety and depression emerge as prevalent issues, significantly impacting the quality of life of individuals and posing substantial challenges for healthcare systems worldwide. With the global rise in mental health disorders, it has become increasingly important to delve deeper into the intricate networks and comorbidities that many individuals experience, especially concerning the major depressive disorder (MDD) and the social anxiety disorder (SAD) [1]. These disorders, sometimes occurring independently but often presenting simultaneously in individuals, have become a focal point in psychiatric research, exploring the avenues of both individual and shared symptoms, triggers, and treatment pathways [2].

SAD is a persistent and severely distressing psychiatric condition, characterized by an enduring fear of being negatively judged in social settings. Although often conflated with mere shyness, which shares certain superficial similarities, SAD is much more debilitating, frequently leading to underdiagnosis and consequently, individuals grappling with its ramifications unnoticed. This disorder can permeate various aspects of an individual’s life, severely affecting academic achievements, hindering social development, destabilizing relationships, and impeding professional success. In recent years, there has been a marked surge in research surrounding anxiety and its allied disorders, largely driven by the adoption and advancement of cognitive-behavioral frameworks. This shift has facilitated a more nuanced understanding of SAD, moving beyond its initial characterization as extreme shyness to a more sophisticated comprehension of the complex underlying mechanisms driving its onset and progression. Moreover, this evolved perspective has been instrumental in devising strategies not merely for its management but also for effectively distinguishing it from more benign conditions, thus paving the way for targeted and effective therapeutic interventions [3–8].

Depression, on the other hand, has seen a significant escalation among young individuals in the recent decade, a trend particularly pronounced in young females. This spike is particularly alarming given that adolescence is a critical stage characterized by swift transitions in social, emotional, and cognitive realms. Depression during this period carries ramifications that extend into various aspects of life, leading to recurrent episodes of depression, triggering other psychiatric conditions, and enduring detriments in relationships, social engagements, education, and career prospects. Consequently, it is of utmost importance to prioritize preventive measures and early interventions for individuals grappling with depression. These strategies conventionally address the precursor elements and manifestations of depression, aiming to mitigate its onset and alleviate its symptoms at the earliest. Individuals from certain backgrounds, including those with a familial history of depression, those experiencing social stressors, such as bullying or tumultuous relationships, those undergoing significant life stresses, and those belonging to particular subgroups, including individuals with chronic health issues or those identifying as part of a sexual minority, find themselves at a heightened risk of developing depression. Recognizable clinical precursors of depression encompass symptoms such as anxiety and heightened irritability. Given the varied manifestations and triggers of depression, it warrants a strategy grounded in indicated and targeted prevention, which has proven to be more effective than universal preventive approaches. In this light, budding interventions rooted in community settings demonstrate potential. Addressing depression demands a nuanced and stepped strategy, beginning with succinct psychosocial interventions, progressing to specialized psychological therapies, and, if necessary, advancing to the prescription of antidepressant medications, paving a pathway for more individualized and effective treatment regimens [9–14].

Delving into the more specific backdrop of MDD and SAD, the comorbidity of SAD in patients with MDD, and MDD in patients with SAD is more common than in the general population, representing a severe global health concern [2]. Studies have shown that approximately half of the patients diagnosed with MDD have an anxiety disorder as a comorbid disease. Among these, social phobia was found to be one of the most common anxiety disorder comorbid with MDD [15]. In various studies conducted globally within the field of social phobia, MDD appears to be the most common comorbid disorder [16, 17]. It has been reported that the association of MDD and SAD was 70%, with SAD usually manifesting before the mood disorder [18]. Common genetic factors suggest a shared background for SAD and MDD. Twin studies also underscore this comorbidity [19]. For patients diagnosed with SAD, it presents as an extremely disabling condition. The severity of symptoms, social disability, and suicide rates indicate that the comorbidity is significantly more serious than “pure” SAD [20]. These studies revealed that SAD comorbidity was associated with early-onset MDD, more frequent depressive episodes, an increased risk of suicide, and higher alcohol consumption [21]. There are limited studies examining the effects of comorbidity on treatment success in SAD [22]. The fact that the presence of SAD comorbidity in patients with MDD is a long-term predictor of poor prognosis further emphasizes the importance of addressing comorbidity [23].

In recent years, there has been an increasing interest in identifying objective markers that can provide insights into the underlying inflammatory processes associated with various psychiatric disorders, including MDD and SAD. Among these, inflammatory biomarkers, such as the neutrophil-to-lymphocyte ratio (NLR), platelet-to-lymphocyte ratio (PLR), monocyte-to-lymphocyte ratio (MLR), neutrophil-to-lymphocyte platelet ratio (NLPR), systemic inflammation index (SII), and systemic inflammation response index (SIRI) have emerged as promising candidates. NLR, PLR, and MLR are relatively simple and cost-effective indices that have been widely studied as potential indicators of systemic inflammation. Elevated levels of these biomarkers have been associated with increased severity and poorer prognosis in various inflammatory and cardiovascular conditions, and they have been recently explored in the context of neuropsychiatric disorders [24]. The NLPR, SII, and SIRI are more comprehensive indices, capturing a broader spectrum of the systemic inflammatory response. Their elevation has been linked with various pathological conditions, underscoring the intricate relationship between inflammation and disease progression [25]. Considering the pivotal role of inflammation in the pathophysiology of neuropsychiatric disorders, these biomarkers can offer valuable insights. Chronic inflammation, as represented by these indices, has been postulated to impact neurotransmitter metabolism, brain function, and ultimately, the clinical presentation of disorders like MDD and SAD [26, 27].

Comorbid SAD is often identified during detailed examinations in routine practice. It is vital to grasp patients’ concerns, understand the impact of anxiety comorbidity on the clinical progression of depression, and strategize the most effective treatments for these individuals. Our study sought to compare the clinical features between patients primarily diagnosed with MDD and secondarily with SAD, against those primarily diagnosed with SAD and secondarily with MDD. Additionally, we aimed to elucidate the potential influence of inflammatory biomarkers in comprehending the nuanced relationship between these coexisting conditions.

## Materials and methods

Our research employed a cross-sectional study design to explore the nuanced relationship between MDD and SAD, utilizing a comprehensive array of inflammatory biomarkers as investigative tools. This approach was built upon reliable and evidence-backed methodology, a decision influenced and supported by a range of pertinent studies [27–31].

### Participants

The study period spanned from December 2021 to June 2022, during which 204 outpatients undergoing routine psychiatric follow-up examinations at the Psychiatry Clinic of Kartal City Hospital were screened. These patients, identified to be in partial remission, were diagnosed according to the International Classification of Diseases, Tenth Revision (ICD-10) criteria.

The division into the MDD–SAD and SAD–MDD groups was predicated on the initial clinical diagnoses documented in the digital health records, complemented by the history obtained directly from the patients. Retrospective analyses were employed to ascertain the onset of social anxiety symptoms, understanding that while early signs of social anxiety were evident owing to subthreshold complaints, the primary clinical presentation and diagnosis were discerned to be MDD in a subset of patients. It should be noted that groups were designated based on the foremost diagnosis at their initial clinical presentation rather than the onset of subtler, earlier symptoms.

The MDD–SAD group comprised 74 individuals primarily diagnosed with MDD as per their clinical presentation, and secondarily with SAD as per the Diagnostic and Statistical Manual of Mental Disorders, Fifth Edition (DSM-5) criteria. Conversely, the SAD–MDD group included 130 individuals who were initially diagnosed with SAD, followed by a secondary diagnosis of MDD.

The inclusion criteria for this study were as follows: participants had to meet the diagnostic criteria for MDD and SAD as outlined in the DSM-5 and be aged between 18 and 65 years.

The exclusion criteria for this study were as follows: the presence of any psychiatric disorder other than MDD and SAD, and having a history of mental retardation, any neurological disease, or head trauma.

### Laboratory data

Values related to laboratory data were obtained from patients’ routine health screenings carried out within the previous six months. Inclusions were made only if these values did not indicate an active infection, and patients did not report any history of infection or clinical condition that might affect blood parameters during that period.

The NLR is a widely recognized measure of systemic inflammation, obtained by dividing the absolute neutrophil count by the absolute lymphocyte count from a complete blood count (CBC). Similarly, the MLR is another metric of inflammation, derived by dividing the absolute monocyte count by the absolute lymphocyte count. The PLR is employed to measure inflammation and immune response, calculated by dividing the absolute platelet count by the lymphocyte count. The NLPR offers an extended view of systemic inflammation, computed by multiplying the NLR by the platelet count. The SII provides a composite view, merging neutrophil, platelet, and lymphocyte counts to capture a broader picture of inflammation. It is calculated as the platelet count multiplied by the NLR. Lastly, the SIRI encompasses multiple cellular components, usually determined as the neutrophil count multiplied by the monocyte count divided by the lymphocyte count.

### Procedure

All participants were asked to complete the following questionnaires:

1. The Beck Depression Inventory (BDI) consisting of 21 questions with a scoring range of 0–3 for each. The severity of depression experienced by individuals is inferred from the total score, with higher scores indicating greater severity [32, 33].
2. The Beck Anxiety Inventory (BAI) developed to measure the severity of anxiety symptoms in psychiatric populations. Each item is scored between 0 and 3, yielding a total score range of 0–63. The higher scores are associated with more severe anxiety symptoms [34, 35].
3. The Liebowitz Social Anxiety Scale (LSAS) developed to evaluate individuals’ social interactions and performance situations where fear and/or avoidance behavior is exhibited. It contains two subscales (fear or anxiety and avoidance) encompassing 24 questions. The score range for each subscale is 24–96, with higher scores indicating more severe social anxiety and avoidance behaviors [36, 37].
4. The Childhood Trauma Questionnaire (CTQ-28), a standardized, retrospective, self-report scale, consisting of 28 items. It yields a total score derived from five subscores concerning childhood sexual, physical, and emotional abuse, as well as emotional and physical neglect [38, 39].
5. Toronto Alexithymia Scale (TAS-20), a 20-item instrument that is one of the most commonly used instruments for measuring alexithymia. Each item is scored on a scale of 1–5, where 1 represents “strongly disagree” and 5 represents “strongly agree.” The TAS-20 encompasses three subscales: difficulty describing feelings, difficulty identifying feelings, and externally oriented thinking subscale [40, 41].

The validity and reliability of all the scales were verified for the Turkish language through comprehensive studies [33–35, 37–39, 41].

### Ethical statement

The study protocol adhered to the ethical guidelines outlined in the 1975 Declaration of Helsinki. Written consent was obtained from each patient participating in the study. The Ethics Committee of the Uskudar University granted approval for this study and all its procedures (date of approval 26/11/2021, ref: 61351342/KASIM 2021-40).

### Statistical analysis

The data for this study was analyzed using the Statistical Package for Social Sciences (SPSS) for Windows, version 22.0. The normal distribution was assessed using the Kolmogorov–Smirnov test along with Skewness–Kurtosis values. Demographic information was analyzed through descriptive statistics. For comparing independent groups with or without normal distribution, the Student’s *t*-test or Mann–Whitney *U*-test was employed. Pearson and Spearman correlation tests were used for correlation analysis of normally or non-normally distributed data. Receiver operating characteristic (ROC) analysis was conducted on inflammatory biomarkers to determine their potential in predicting distinctions between the MDD–SAD and SAD–MDD groups. A *P* value of < 0.05 was considered statistically significant.

## Results

Our study encompassed a total of 204 patients, 74 diagnosed primarily with MDD and comorbid SAD (MDD–SAD), and 130 diagnosed primarily with SAD and comorbid MDD (SAD–MDD). A detailed comparison of their sociodemographic and clinical features, including scale scores from BDI, BAI, TAS-20, CTQ-28, and LSAS, is delineated in Tables 1 and 2.

**Table 1:** Comparison of sociodemographic characteristics and clinical features between the MDD–SAD group and the SAD–MDD group

		**MDD–SAD group** **(*n* ═ 74)**	**SAD–MDD group** **(*n* ═ 130)**	***P* value**
Sex	Female	44 (59.5)	38 (29.2)	0.001
	Male	30 (40.5)	92 (70.8)	
Marital status	Married	34 (45.9)	24 (18.5)	0.001
	Single/divorced	40 (54.1)	106 (81.5)	
Age at initial presentation of SAD symptoms	< 15 years	56 (75.7)	58 (44.6)	0.001
	≥ 15 years	18 (24.3)	72 (55.4)	
Onset of SAD during a depressive episode	Yes	14 (18.9)	52 (40.0)	0.002
	No	60 (81.1)	78 (60.0)	
Depressive episodes with atypical features	Yes	56 (75.7)	76 (58.5)	0.010
	No	18 (24.3)	54 (41.5)	
Complete remission in between depressive episodes	Yes	68 (91.9)	92 (70.8)	0.001
	No	6 (8.1)	38 (29.2)	
Somatoform disorder except conversion disorder	Yes	10 (13.5)	0 (0.0)	0.001
	No	64 (86.5)	130 (100.0)	
Age (years)		26.9 ± 8.2	27.9 ± 5.6	0.304
Education (years)		8.7 ± 3.1	12.4 ± 2.7	0.001
Number of suicide attempts		2.3 (0 – 5)	2 (0 – 3)	0.090
Age at initial presentation of SAD symptoms		12.2 ± 2.9	14.9 ± 3.3	0.001
Duration of SAD (years)		14.7 ± 8.4	13.4 ± 4.9	0.251
Number of depressive episodes with seasonal pattern		2.9 (0 – 8)	4.8 (0 – 13)	0.001
Number of depressive episodes		5.9 (0 – 11)	6.7 (0 – 15)	0.335

The numbers represent *n* (%), mean ± standard deviation or median (minimum – maximum). MDD: Major depressive disorder; SAD: Social anxiety disorder.

**Table 2:** Comparison of the scales scores between the MDD–SAD group and the SAD–MDD group

	**MDD–SAD group**	**SAD–MDD group**	***P* values**
BDI	26.5 ± 9.8	21.9 ± 10.8	0.003
BAI	28.9 ± 9.4	26.0 ± 11.2	0.062
LSAS – Fear or anxiety	65.2 ± 7.6	67.4 ± 11.1	0.102
LSAS – Avoidance	63.0 ± 10.4	64.6 ± 11.3	0.325
TAS-20 – Difficulty identifying feelings	21.9 ± 5.2	18.9 ± 5.4	0.001
TAS-20 – Difficulty describing feelings	16.5 ± 3.7	15.8 ± 3.7	0.231
TAS-20 – Externally-oriented thinking	22.5 ± 3.8	22.1 ± 3.8	0.503
CTQ-28 – Physical neglect	6.5 ± 0.8	6.0 ± 1.3	0.001
CTQ-28 – Emotional neglect	13.6 ± 5.2	12.2 ± 4.2	0.039
CTQ-28 – Physical abuse	5.2 (5 – 14)	5.0 (5 – 10)	0.010
CTQ-28 – Emotional abuse	8.7 (5 – 19)	7.7 (5 – 14)	0.010
CTQ-28 – Sexual abuse	5.0 (5 – 19)	5.0 (5 – 9)	0.002

The numbers represent mean ± standard deviation or median (minimum – maximum). MDD: Major depressive disorder; SAD: Social anxiety disorder; BDI: Beck Depression Inventory; BAI: Beck Anxiety Inventory; LSAS: Liebowitz Social Anxiety Scale; TAS-20: Toronto Alexithymia Scale; CTQ-28: Childhood Trauma Questionnaire.

Interestingly, our results suggest that atypical depressive features were more frequently observed in the MDD–SAD group (75.7%) compared to the SAD–MDD group (58.5%) (*P* ═ 0.010).

In terms of scale scores (Table 2), the MDD–SAD group presented higher scores on the BDI (26.5 ± 9.8 vs 21.9 ± 10.8; *P* ═ 0.003), indicating more severe depressive symptoms. They also scored significantly higher on the CTQ-28 subcategories, such as physical neglect and emotional neglect (*P* < 0.05).

The correlation analysis (Table 3) demonstrated significant relationships between several scale scores in both groups.

**Table 3:** Correlation analysis between scales scores within the MDD–SAD group and the SAD–MDD group

	**MDD–SAD group**		**SAD–MDD group**	
	**BDI**	**BAI**	**BDI**	**BAI**
LSAS – Fear or Anxiety	0.326**	0.394**	0.205*	0.201*
LSAS – Avoidance	0.246*	0.231*	0.233**	0.118
TAS-20 – Difficulty identifying feelings	0.334**	0.084	0.192*	0.166
TAS-20 – Difficulty describing feelings	0.030	0.044	0.083	0.078
TAS-20 – Externally-oriented thinking	0.009	0.074	0.077	0.014
CTQ-28 – Physical neglect	0.081	0.187	0.019	0.219*
CTQ-28 – Emotional neglect	0.311**	0.243*	0.172	0.298**
CTQ-28 – Physical abuse	0.157	0.116	0.079	0.209*
CTQ-28 – Emotional abuse	0.163	0.265*	0.153	0.199*
CTQ-28 – Sexual abuse	0.277*	0.050	0.200*	0.198*

*P < 0.05; **P < 0.01. MDD: Major depressive disorder; SAD: Social anxiety disorder; BDI: Beck Depression Inventory; BAI: Beck Anxiety Inventory; LSAS: Liebowitz Social Anxiety Scale; TAS-20: Toronto Alexithymia Scale; CTQ-28: Childhood Trauma Questionnaire.

The inflammatory biomarkers were analyzed and compared between the MDD–SAD and SAD–MDD groups. Notable distinctions emerged across various indices, as presented in Table 4. The MDD–SAD group consistently exhibited higher values across inflammatory biomarkers, including NLR, NLPR, SII, and SIRI, when compared to the SAD–MDD group.

**Table 4:** Comparison of the inflammatory biomarkers between the MDD–SAD and SAD–MDD groups

	**MDD–SAD group** **(*n* ═ 74)**	**SAD–MDD group** **(*n* ═ 130)**	***P* value**
NLR	2.48 (0.70 – 6.14)	1.73 (0.53 – 5.94)	0.002
MLR	0.31 (0.09 – 3.53)	0.27 (0.09 – 1.02)	0.060
PLR	167 (53 – 494)	144 (47 – 537)	0.109
NLPR	0.010 (0.001 – 0.030)	0.008 (0.001 – 0.030)	0.004
SII	608.6 (164.7 – 2421.2)	471.1 (143.1 – 2403.9)	0.002
SIRI	1131.5 (155.2 – 17294.1)	815.5 (155.2 – 3685.9)	0.003

The numbers represent the median (minimum – maximum) values. MDD: Major depressive disorder; SAD: Social anxiety disorder; NLR: Neutrophil-to-lymphocyte ratio; MLR: Monocyte-to-lymphocyte ratio; PLR: Platelet-to-lymphocyte ratio; NLPR: Neutrophil-to-lymphocyte platelet ratio; SII: Systemic inflammation index; SIRI: Systemic inflammation response index.

**Figure 1.**
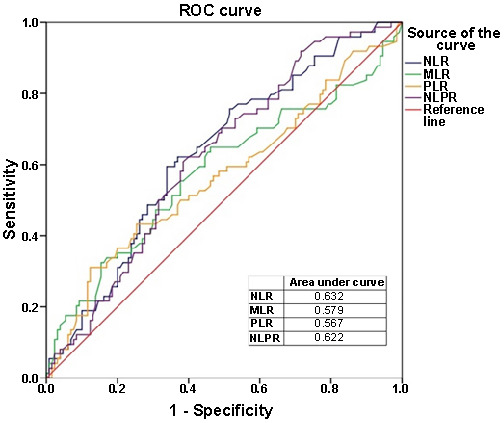
**ROC curves for NLR, MLR, PLR, and NLPR evaluating their efficiency in distinguishing between the MDD–SAD and SAD–MDD groups.** ROC: Receiver operating characteristic; NLR: Neutrophil-to-lymphocyte ratio; MLR: Monocyte-to-lymphocyte ratio; PLR: Platelet-to-lymphocyte ratio; NLPR: Neutrophil-to-lymphocyte platelet ratio; MDD: Major depressive disorder; SAD: Social anxiety disorder.

**Figure 2.**
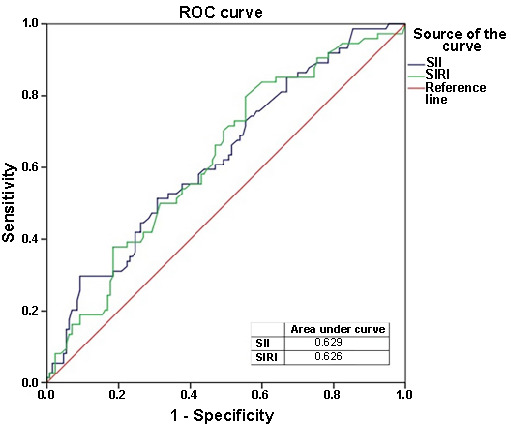
**ROC curves for SII and SIRI evaluating their efficiency in distinguishing between the MDD–SAD and SAD–MDD groups.** ROC: Receiver operating characteristic; SII: Systemic inflammation index; SIRI: Systemic inflammation response index; MDD: Major depressive disorder; SAD: Social anxiety disorder.

An ROC analysis of various inflammatory biomarkers, evaluating their efficiency in distinguishing between the MDD–SAD and SAD–MDD groups is presented in Table 5. In summary, as presented, these inflammatory biomarkers exhibit moderate discriminative ability in distinguishing between the MDD–SAD and SAD–MDD groups (Figures 1 and 2).

**Table 5:** An ROC analysis of various inflammatory biomarkers, evaluating their efficiency in distinguishing between the MDD–SAD and SAD–MDD groups

	**AUC**	***P* value**	**Lower bound**	**Upper bound**	**Cut-off point**	**Sensitivity** **(%)**	**Specificity** **(%)**
**NLR**	0.632	0.002	0.555	0.710	2.01	62.2	62.3
**MLR**	0.579	0.060	0.494	0.665			
**PLR**	0.567	0.109	0.483	0.652			
**NLPR**	0.622	0.004	0.545	0.699	0.009	60.8	61.5
**SII**	0.629	0.002	0.550	0.707	520.0	58.1	57.7
**SIRI**	0.626	0.003	0.548	0.705	1000.6	56.8	56.9

ROC: Receiver operating characteristic; AUC: Area under the curve; MDD: Major depressive disorder; SAD: Social anxiety disorder; NLR: Neutrophil-to-lymphocyte ratio; MLR: Monocyte-to-lymphocyte ratio; PLR: Platelet-to-lymphocyte ratio; NLPR: Neutrophil-to-lymphocyte platelet ratio; SII: Systemic inflammation index; SIRI: Systemic inflammation response index.

## Discussion

Our study revealed several noteworthy findings. The most prominent observation was the earlier onset of SAD in patients primarily diagnosed with MDD (75.7% vs 44.6%; *P* ═ 0.001). These results contribute novel insights into the understanding of the temporal relationship between SAD and MDD, underscoring the importance of early intervention strategies in this population. Additionally, a higher prevalence of the female gender was observed in the MDD–SAD group (59.5% vs 29.2%; *P* ═ 0.001). This interesting finding lends support to previous studies indicating a varying gender distribution in SAD and MDD, as well as their comorbidity [20–23]. Notably, we discovered that depressive complaints in this group were not only more severe but also displayed an atypical pattern (75.7% vs 58.5%; *P* ═ 0.010), aligning with the complex presentations often seen in patients with SAD. In evaluating the inflammatory biomarkers between the MDD–SAD and SAD–MDD groups, significant differences were identified in the NLR (2.48 vs 1.73; *P* ═ 0.002), NLPR (0.010 vs 0.008; *P* ═ 0.004), SII (608.6 vs 471.1; *P* ═ 0.002), and SIRI (1131.5 vs 815.5; *P* ═ 0.003). The NLR showcased a cut-off point of 2.01 with a sensitivity of 62.2% and a specificity of 62.3% for distinguishing between MDD–SAD and SAD–MDD groups. The NLPR exhibited a cut-off point of 0.009 with a sensitivity of 60.8% and a specificity of 61.5%. The SII was characterized by a cut-off point of 520.0, a sensitivity of 58.1%, and a specificity of 57.7%. Lastly, the SIRI presented a cut-off point of 1000.6, achieving a sensitivity of 56.8% and a specificity of 56.9%.

The comorbidity between MDD and SAD is notably high [42–45]. In the present study, we utilized the initial clinical diagnoses documented in the digital health records, complemented by patients’ anamneses, to classify individuals into the MDD–SAD and SAD–MDD groups. It is pertinent to acknowledge that this classification, dictated predominantly by the diagnoses made during initial clinical consultations, may not precisely reflect the true onset of subtler symptoms experienced by the patients throughout their illness trajectories. A deeper investigation into the onset and progression of these illnesses, potentially distinguishing between comorbidities and post-morbidities, might provide a more nuanced understanding of the interplay between MDD and SAD. Although we adhered to the primary diagnoses recorded at the initial point of clinical intervention, we recognize that this approach might not fully encapsulate the complex nature of onset patterns in comorbid MDD and SAD.

The influence of early life experiences on the development of both SAD and MDD is well documented. People begin social interaction early in life, and the manifestation of anxiety and avoidance could spiral into a vicious cycle of social skills regression, escalated anxiety, and diminished self-esteem [46]. Interestingly, our results suggest that this relationship might be bidirectional—SAD could also follow after depression. This is particularly relevant in individuals whose social lives are disrupted by a depressive episode, as the subsequent feelings of guilt and worthlessness could potentially trigger the development of SAD.

Our data showed a predominance of single or divorced individuals within the SAD–MDD group, with a rate of 81.5%. This rate is significantly higher when compared to the 54.4% observed in the MDD–SAD group. This observation is consistent with previous studies, which reported that SAD patients often live alone are unmarried or divorced. The potential influence of social support networks on hospital admissions is a significant consideration in light of these findings [47].

We found high mean scores for emotional neglect in our sample, with the MDD–SAD group exhibiting significantly higher scores for both physical and emotional neglect. Additionally, this group demonstrated an earlier onset age of SAD. These findings suggest that emotional neglect and abuse are significant factors contributing to the development of SAD, reinforcing the findings of previous studies [48]. Within the context of trauma, our study revealed that trauma sub-scores correlated with anxiety levels, particularly in patients primarily diagnosed with SAD. This correlation indicates that trauma-focused psychotherapeutic approaches may hold promise for treating anxiety in patients with comorbidities.

Regarding the early onset of anxiety symptoms in the MDD–SAD group, it is crucial to highlight that the initial manifestations of SAD can be subtle and may sometimes be easily overlooked or misconstrued, which could delay a formal diagnosis until the appearance of more pronounced depressive symptoms. Moreover, it was observed that upon being introduced to the DSM-5 criteria for SAD, patients could retrospectively identify earlier onset of social anxiety symptoms, albeit at a subclinical level. These revelations shed light on a critical dimension of mental health diagnostics, emphasizing the necessity to consider the dynamic interrelation of symptoms that evolve over time, sometimes in a non-linear and overlapping manner, which adds a significant layer of complexity in determining the primary disorder. Moving forward, it behooves researchers and clinicians alike to adopt a more nuanced approach in diagnosing, perhaps incorporating a meticulous analysis of early life symptoms and their progression to comprehend the multifaceted relationship between SAD and MDD more fully.

In our study, the score for the difficulty identifying feelings subscale was higher in the MDD group than in the SAD group, as difficulty identifying feelings emerges as an important predictor of psychopathology in patients with MDD [49–52]. On the other hand, there was a significant correlation between depression levels and difficulty identifying feelings sub-scores in both patient groups, the MDD–SAD group and the SAD–MDD group.

Atypical depressive episodes were more prevalent in the MDD–SAD group, echoing findings in existing literature [53]. Both SAD and atypical depression share features such as interpersonal rejection sensitivity. This commonality might provide a possible explanation for the high prevalence of atypical depressive episodes in this group.

Our study suggests a more pronounced remission of depressive episodes in cases initially presenting with SAD and later developing MDD, a phenomenon potentially indicative of “reactive depression.” This nuanced interrelation raises pivotal questions regarding the developmental sequence and interdependence of these disorders. The findings underscore the necessity for clinicians to identify SAD to forestall subsequent MDD manifestations, paving the path for more effective therapeutic interventions [54–57].

Central to our investigation’s findings is the noteworthy elevation of inflammatory biomarkers in the MDD–SAD group. Distinctly elevated levels of NLR, NLPR, SII, and SIRI were discerned in this cohort relative to the SAD–MDD group. Such findings amplify the discourse surrounding the intricate relationship between inflammation and neuropsychiatric disorders. Chronic inflammation, as represented by these biomarkers, is increasingly postulated to play a transformative role in the pathophysiology of psychiatric conditions, particularly MDD [31, 58, 59].

The pronounced presence of systemic inflammation within the MDD–SAD cohort offers a compelling lens through which to interpret the heightened severity and atypical manifestations of their depressive symptoms. It is plausible that the inflammatory milieu might not just be a consequence, but a driving force behind the observed heightened clinical severity. Previous studies have highlighted the role of inflammation in neuroplasticity, neurotransmitter metabolism, and even the brain-gut axis, providing a framework for understanding the biochemical foundations of psychiatric symptomatology [27, 60].

Furthermore, the intricate interplay between social stressors, such as emotional neglect or trauma, and the body’s inflammatory response presents an area ripe for exploration. Could early life adversities, which were prevalent in our MDD–SAD group, serve as initial triggers, igniting the inflammatory cascade and subsequently setting the stage for psychiatric manifestations?

Given this landscape, it becomes paramount to consider anti-inflammatory strategies as adjunctive therapies in the holistic management of comorbid MDD and SAD. Tailoring treatments to address not only the manifested psychological symptoms but also the underlying inflammatory state might usher in a paradigm shift in how we approach such complex comorbidities, potentially improving both clinical outcomes and patients’ quality of life. Given the frequent comorbidity of depression and anxiety disorders, it is imperative that this factor is taken into account during treatment planning. Focusing on a single disorder could hinder overall treatment success.

This study offers valuable real-life data by comparing the clinical and sociodemographic characteristics of patients with a primary diagnosis of MDD and a co-diagnosis of SAD to those with a primary diagnosis of SAD and a co-diagnosis of MDD. However, our research bears several limitations. Being a single-center study, it encompasses a relatively small sample size. Data collection was reliant on self-report questionnaires and given that the study population comprised depressive patients actively seeking treatment, depressive symptoms might have been reported with heightened severity. This aspect potentially limits the generalizability of our findings to patients who are not undergoing treatment. Furthermore, due to the inherent nature of their anxiety disorder, SAD patients may not have fully conveyed their issues and experiences. It is also important to note that the laboratory parameters were obtained from patients who were not drug-naive, potentially influencing the biomarker levels. There was an absence of detailed data on the chronicity and specific duration of the disorders in the MDD–SAD and SAD–MDD groups. Given that the severity and impact of both MDD and SAD can vary significantly over time and can be influenced by the chronicity of the symptoms, future studies should aim to incorporate this variable for a more nuanced understanding. The modest significance in the differences of biomarker values identified in this study necessitates a cautious interpretation of the results. Expanding the study to include a larger sample size could potentially yield more robust findings. The exclusion of a healthy control group to benchmark these observations is a notable limitation. Future endeavors in this research area should contemplate a design inclusive of a control group comprised of healthy individuals to facilitate a more comprehensive analysis and understanding of the biomarker variations in individuals with MDD–SAD and SAD–MDD comorbidity. The discrepancy in the sample sizes might have introduced a bias in the conducted comparative and correlational analyses. Lastly, the cross-sectional design of our study hinders the elucidation of causal relationships. Future longitudinal studies, employing larger clinical samples, promise to yield more comprehensive insights.

This study underscores several critical clinical implications. Foremost, given the earlier onset of SAD in patients primarily diagnosed with MDD, it is imperative for healthcare providers to be proactive in assessing signs of SAD, especially in younger patients diagnosed with MDD. The distinct over-representation of females in the MDD–SAD group necessitates consideration for gender-sensitive therapeutic approaches. This might involve tailored interventions specifically designed for women. Furthermore, the notable connection between trauma sub-scores and anxiety levels, especially in patients primarily diagnosed with SAD, points towards the potential efficiency of trauma-focused psychotherapeutic methods for treating anxiety in these patients. Additionally, this study shines a light on the potential vulnerability of single or divorced individuals, suggesting the pivotal role of a strong social support system. The absence of such support might act as a risk factor or it might potentially intensify the severity of these disorders. This highlights the need for interventions that either bolster social support or aid in coping with its absence. Moreover, the prominence of atypical depressive episodes in the MDD–SAD group signifies the necessity for clinicians to recognize and adeptly treat this subtype of depression, especially when it coexists with SAD. Notably, the MDD–SAD group’s elevated inflammatory biomarkers and frequent presentation of atypical depressive episodes underscore the importance for clinicians to be adept at identifying and managing this depression subtype, especially when it is concurrent with SAD. Such elevated biomarkers signify a heightened inflammatory state, suggesting a potential role of inflammation in exacerbating symptoms or even as a contributing etiological factor. Overall, these findings advocate for a holistic approach in treating patients with comorbid MDD and SAD, emphasizing the simultaneous management of both conditions for optimal patient outcomes.

## Conclusion

In conclusion, our findings shed light on the intricate relationship between SAD and MDD, emphasizing the early, perhaps subthreshold onset of SAD symptoms in patients primarily diagnosed with MDD. Despite the early onset of SAD symptoms, many patients initially seek help for depressive symptoms, underscoring the pivotal role of early SAD diagnosis and treatment in effectively managing such comorbidities and potentially averting more severe depressive episodes. The manifestation of more severe and atypical depressive symptoms, a pronounced history of trauma, and a higher ratio of females in the MDD–SAD group corroborate the complexity of these intertwined conditions. Furthermore, the study suggests a potential involvement of elevated inflammatory biomarkers in the pathophysiology of these comorbid disorders, encouraging a deeper exploration of the underlying mechanisms in future research. As we move forward, it becomes imperative to decipher the temporal dynamics between SAD and MDD with a keen focus on gender-specific presentations and the role of trauma, all while adhering to the DSM-5 diagnostic criteria to foster nuanced understandings and enhanced therapeutic approaches.

## References

[1] Ibbad S, Baig LA, Ahmer Z, Shahid F (2022 Mar-Apr). Prevalence of anxiety and depression in high school students of Karachi, Pakistan. Pak J Med Sci.

[2] Kazgan Kılıçaslan A, Yıldız S, Kurt O, Atmaca M (2022 Sep 1). Personality characteristics, anxiety sensitivity, anxiety, and depression levels on patients diagnosed with psychogenic pruritus. Alpha Psychiatry.

[3] Alomari NA, Bedaiwi SK, Ghasib AM, Kabbarah AJ, Alnefaie SA, Hariri N (2022). Social anxiety disorder: associated conditions and therapeutic approaches. Cureus.

[4] Leichsenring F, Leweke F (2017). Social anxiety disorder. N Engl J Med.

[5] Guo S, Deng W, Wang H, Liu J, Liu X, Yang X (2021). The efficacy of internet-based cognitive behavioural therapy for social anxiety disorder: a systematic review and meta-analysis. Clin Psychol Psychother.

[6] Hofmann SG, Anu Asnaani MA, Hinton DE (2010). Cultural aspects in social anxiety and social anxiety disorder. Depress Anxiety.

[7] Morrison AS, Heimberg RG (2013). Social anxiety and social anxiety disorder. Ann Rev Clin Psychol.

[8] Chen J, Short M, Kemps E (2020). Interpretation bias in social anxiety: a systematic review and meta-analysis. J Affect Disord.

[9] Thapar A, Eyre O, Patel V, Brent D (2022). Depression in young people. Lancet.

[10] Loades ME, Midgley N, Herring GT, O’Keeffe S, Goodyer IM, Barrett B (2023). In context: lessons about adolescent unipolar depression from the improving mood with psychoanalytic and cognitive therapies trial. J Am Acad Child Adolesc Psychiatry.

[11] Huang XC, Zhang YN, Wu XY, Jiang Y, Cai H, Deng YQ (2023). A cross-sectional study: family communication, anxiety, and depression in adolescents: the mediating role of family violence and problematic internet use. BMC Public Health.

[12] Bertha EA, Balázs J (2013 Oct). Subthreshold depression in adolescence: a systematic review. Eur Child Adolesc Psychiatry.

[13] Sheets ES, Craighead WE (2014 Dec 1). Comparing chronic interpersonal and noninterpersonal stress domains as predictors of depression recurrence in emerging adults. Behav Res Ther.

[14] Cui R (2015). A systematic review of depression. Curr Neuropharmacol.

[15] De la Torre-Luque A, Essau CA (2019). Symptom network connectivity in adolescents with comorbid major depressive disorder and social phobia. J Affect Disord.

[16] Faravelli C, Zucchi T, Viviani B, Salmoria R, Perone A, Paionni A (2000). Epidemiology of social phobia: a clinical approach. Eur Psychiatry.

[17] Karlsson B, Sigström R, Östling S, Waern M, Börjesson-Hanson A, Skoog I (2016). DSM-IV and DSM-5 prevalence of social anxiety disorder in a population sample of older people. Am J Geriatr Psychiatry.

[18] Koyuncu A, Ince E, Ertekin E, Tükel R (2019). Comorbidity in social anxiety disorder: diagnostic and therapeutic challenges. Drugs Context.

[19] Weissman MM (1988). The epidemiology of anxiety disorders: rates, risks and familial patterns. J Psychiatr Res.

[20] Lecrubier Y, Weiller E (1997). Comorbidities in social phobia. Int Clin Psychopharmacol.

[21] Rozen N, Gilboa-Schechtman E, Marom S, Hermesh H, Aderka IM (2022). Comorbid major depressive disorder in cognitive-behavior group treatment for social anxiety disorder: an examination of processes of symptom change. Psychotherapy.

[22] Van Velzen CJ, Emmelkamp PM, Scholing A (1997). The impact of personality disorders on behavioral treatment outcome for social phobia. Behav Res Ther.

[23] Buckman JEJ, Underwood A, Clarke K, Saunders R, Hollon SD, Fearon P (2018 Aug). Risk factors for relapse and recurrence of depression in adults and how they operate: a four-phase systematic review and meta-synthesis. Clin Psychol Rev.

[24] Bulut NS, Yorguner N, Carkaxhiu Bulut G (2021). The severity of inflammation in major neuropsychiatric disorders: comparison of neutrophil–lymphocyte and platelet–lymphocyte ratios between schizophrenia, bipolar mania, bipolar depression, major depressive disorder, and obsessive compulsive disorder. Nordic J Psychiatry.

[25] Ünal Ç, Tunçer G, Çopur B, Pilanci KN, Okutur KS, Yararbaş K (2023). Clinical and inflammation marker features of cancer patients with COVID-19: data of Istanbul, Turkey multicenter cancer patients (2020–2022). Curr Med Res Opin.

[26] Marazziti D, Torrigiani S, Carbone MG, Mucci F, Flamini W, Ivaldi T (2022). Neutrophil/lymphocyte, platelet/lymphocyte, and monocyte/lymphocyte ratios in mood disorders. Curr Med Chem.

[27] Amitai M, Kaffman S, Kroizer E, Lebow M, Magen I, Benaroya-Milshtein N (2022). Neutrophil to-lymphocyte and platelet-to-lymphocyte ratios as biomarkers for suicidal behavior in children and adolescents with depression or anxiety treated with selective serotonin reuptake inhibitors. Brain Behav Immun.

[28] Mazza MG, Lucchi S, Tringali AGM, Rossetti A, Botti ER, Clerici M (2018). Neutrophil/lymphocyte ratio and platelet/lymphocyte ratio in mood disorders: a meta-analysis. Prog Neuropsychopharmacol Biol Psychiatry.

[29] Su M, Ouyang X, Song Y (2022 Jul 1). Neutrophil to lymphocyte ratio, platelet to lymphocyte ratio, and monocyte to lymphocyte ratio in depression: a meta-analysis. J Affect Disord.

[30] Meng F, Yan X, Qi J, He F (2022 Oct 15). Association of neutrophil to lymphocyte ratio, platelet to lymphocyte ratio, and monocyte to lymphocyte ratio with depression: a cross-sectional analysis of the NHANES data. J Affect Disord.

[31] Shan M, Yang Z, Sun Z, Yang Y, Cheng Q, Pan Y (2023 Sep 13). Association between platelet to lymphocyte ratio and depression and symptom severity among adults in the United States: a cross-sectional study. Heliyon.

[32] Beck AT, Steer RA, Carbin MG (1988). Psychometric properties of the Beck depression inventory: twenty-five years of evaluation. Clin Psychol Rev.

[33] Hisli N (1988). A study on the validity of Beck depression inventory. Turk Psikol Derg.

[34] Beck AT, Steer RA (1991). Relationship between the Beck anxiety inventory and the Hamilton anxiety rating scale with anxious outpatients. J Anxiety Disord.

[35] Ulusoy M, Şahin N, Erkman H (1998). Turkish version of the Beck anxiety inventory: psychometric properties. J Cogn Psychother.

[36] Heimberg RG, Horner KJ, Juster HR, Safren SA, Brown EJ, Schneier FR (1999). Psychometric properties of the Liebowitz social anxiety scale. Psychol Med.

[37] Soykan Ç, Özgüven HD, Gençöz T (2003). Liebowitz social anxiety scale: the Turkish version. Psychol Rep.

[38] Scher CD, Stein MB, Asmundson GJ, McCreary DR, Forde DR (2001). The childhood trauma questionnaire in a community sample: psychometric properties and normative data. J Trauma Stress.

[39] Şar V, Öztürk E, İkikardeş E (2012). Çocukluk Çaği Ruhsal Travma Ölçeğinin Türkçe uyarlamasinin geçerlilik ve güvenilirliği. Türkiye Klinikleri J Med Sci.

[40] Kooiman CG, Spinhoven P, Trijsburg RW (2002). The assessment of alexithymia: a critical review of the literature and a psychometric study of the Toronto Alexithymia Scale-20. J Psychosom Res.

[41] Güleç H, Köse S, Güleç MY, Çitak S, Evren C, Borckardt J (2009). Reliability and factorial validity of the Turkish version of the 20-item Toronto Alexithymia Scale (TAS-20). Klin Psikofarmakol Bul.

[42] Brown TA, Campbell LA, Lehman CL, Grisham JR, Mancill RB (2001). Current and lifetime comorbidity of the DSM-IV anxiety and mood disorders in a large clinical sample. J Abnorm Psychol.

[43] Kalsoom U (2019). Gender role in anxiety, depression and quality of life in chronic kidney disease patients: anxiety, depression and QOL in chronic kidney diseases patients. Pak J Med Sci.

[44] Salk RH, Hyde JS, Abramson LY (2017). Gender differences in depression in representative national samples: meta–analyses of diagnoses and symptoms. Psychol Bull.

[45] Tang X, Liu Q, Cai F, Tian H, Shi X, Tang S (2022). Prevalence of social anxiety disorder and symptoms among Chinese children, adolescents and young adults: a systematic review and meta-analysis. Front Psychol.

[46] Ebesutani C, Fierstein M, Viana AG, Trent L, Young J, Sprung M (2015). The role of loneliness in the relationship between anxiety and depression in clinical and school-based youth. Psychol Sch.

[47] Dilbaz N, Güz H (2006). Sosyal anksiyete bozukluğunun fenomenolojisi; anksiyete bozukluklari. Ankara: TPD Yayınevi;.

[48] Acartürk C, de Graaf R, Van Straten A, Have MT, Cuijpers P (2008). Social phobia and number of social fears, and their association with comorbidity, health-related quality of life and help seeking: a population-based study. Soc Psychiatry Psychiatr Epidemiol.

[49] Li S, Zhang B, Guo Y, Zhang J (2015). The association between alexithymia as assessed by the 20-item Toronto Alexithymia Scale and depression: a meta-analysis. Psychiatry Res.

[50] Sagar R, Talwar S, Desai G, Chaturvedi SK (2021). Relationship between alexithymia and depression: a narrative review. Indian J Psychiatry.

[51] Günther V, Rufer M, Kersting A, Suslow T (2016). Predicting symptoms in major depression after inpatient treatment: the role of alexithymia. Nordic J Psychiatry.

[52] Jonsson G, Franzén L, Nyström MB,  Davis PA (2020). Integrating yoga with psychological group-treatment for mixed depression and anxiety in primary healthcare: an explorative pilot study. Complement Ther Clin Pract.

[53] Brailean A, Curtis J, Davis K, Dregan A, Hotopf M (2020). Characteristics, comorbidities, and correlates of atypical depression: evidence from the UK Biobank mental health survey. Psychol Med.

[54] Showraki M (2019). Reactive depression: lost in translation!. J Nervous Ment Dis.

[55] Smith CE, Leenerts MH, Gajewski BJ (2003). A systematically tested intervention for managing reactive depression. Nurs Res.

[56] Benjaminsen S (1981). Primary non-endogenous depression and features attributed to reactive depression. J Affect Disord.

[57] Willner P, Wilkes M, Orwin A (1990). Attributional style and perceived stress in endogenous and reactive depression. J Affect Disord.

[58] Demir S, Atli A, Bulut M, İbiloğlu AO, Güneş M, Kaya M (2015). Neutrophil–lymphocyte ratio in patients with major depressive disorder undergoing no pharmacological therapy. Neuropsychiatr Dis Treat.

[59] Cheng Y, Wang Y, Wang X, Jiang Z, Zhu J, Fang S (2022). Neutrophil-to-lymphocyte ratio, platelet-to-lymphocyte ratio, and monocyte-to-lymphocyte ratio in depression: an updated systematic review and meta-analysis. Front Psychiatry.

[60] Ekinci O, Ekinci A (2017). The connections among suicidal behavior, lipid profile and low-grade inflammation in patients with major depressive disorder: a specific relationship with the neutrophil-to-lymphocyte ratio. Nordic J Psychiatry.

